# Effect of aspirin use on conversion risk from mild cognitive impairment to Alzheimer’s disease

**DOI:** 10.3389/fnagi.2025.1603892

**Published:** 2025-08-06

**Authors:** Bo Kyu Choi, Yeonju Jin, Hokyung Lee, Sung-Woo Kim, Sojeong Park, Ickpyo Hong, Min Seok Baek

**Affiliations:** ^1^Department of Neurology, Gangnam Severance Hospital, Yonsei University College of Medicine, Seoul, Republic of Korea; ^2^Department of Occupational Therapy, Graduate School, Yonsei University, Wonju, Republic of Korea; ^3^Department of Neurology, Wonju Severance Christian Hospital, Yonsei University Wonju College of Medicine, Wonju, Republic of Korea; ^4^Research Institute of Metabolism and Inflammation, Wonju, Republic of Korea; ^5^Department of Biostatistics and Computing, Yonsei University, Seoul, Republic of Korea; ^6^Department of Occupational Therapy, College of Software and Digital Healthcare Convergence, Yonsei University, Wonju, Republic of Korea

**Keywords:** mild cognitive impairment, Alzheimer’s disease, dementia, aspirin, nationwide claim data

## Abstract

**Background:**

The potential effect of the antiplatelet and anti-inflammatory properties of aspirin on Alzheimer’s disease development, especially its role in the progression from mild cognitive impairment to Alzheimer’s disease dementia, remains controversial. To evaluate the association between aspirin, use and the risk of conversion to Alzheimer’s disease dementia among individuals diagnosed with mild cognitive impairment.

**Methods:**

In this retrospective population-based cohort study, we used the Korean National Health Insurance Service database to collect data on patients with mild cognitive impairment enrolled between 2013 and 2016 and followed up until 2021. In total, 508,107 patients initially diagnosed with mild cognitive impairment (192,538 with aspirin prescriptions and 315,569 without aspirin prescriptions) were enrolled. Aspirin use was assessed by extracting information from the Korean National Health Insurance Service database using aspirin prescription codes. The primary outcome was newly diagnosed Alzheimer’s disease dementia. Hazard ratios and 95% confidence intervals for Alzheimer’s disease were analyzed according to aspirin use using Cox proportional hazards regression analysis. Secondary outcomes included ischemic and hemorrhagic stroke risk associated with aspirin use.

**Results:**

The data of 508,107 individuals were analyzed (mean [standard deviation] age, 67.6 [10.7] years; 66.8% women and 33.2% men), and 39,318 developed Alzheimer’s disease (22,572 controls and 16,746 using aspirin). The rate of conversion to Alzheimer’s disease was lower in the aspirin user group, and the time to Alzheimer’s disease dementia occurrence was longer than in the nonuser group. A decreased Alzheimer’s disease dementia risk was found in patients using aspirin in Model 2 (adjusted hazard ratio, 0.939; 95% confidence interval, 0.920–0.959), with more pronounced effects in individuals aged ≥65 years (Model 2 adjusted hazard ratio, 0.934; 95% confidence interval, 0.914–0.955). For hemorrhagic stroke, the risk increased with aspirin use across all age groups, with the highest risk observed in younger patients (Model 2 adjusted hazard ratio, 5.082; 95% confidence interval, 4.838–5.338).

**Conclusion:**

Aspirin use was associated with reduced Alzheimer’s disease risk in older patients with mild cognitive impairment. Notably, the bleeding risk associated with aspirin use should be considered, and personalized treatment should be provided.

## 1 Introduction

Alzheimer’s disease (AD) is the most common cause of dementia and is characterized by progressive cognitive and functional decline. It represents a significant public health challenge owing to its rising prevalence in aging populations and substantial societal and economic burdens (Livingston et al., 2020). Mild cognitive impairment (MCI), a transitional phase between normal aging and dementia, represents a critical stage in which timely intervention may delay or prevent the progression to dementia, offering substantial clinical and societal benefits (Mitchell and Shiri-Feshki, 2009; Dunne et al., 2021).

Aspirin, widely used for its antiplatelet and anti-inflammatory properties, has been proposed as a potential intervention for mitigating cognitive decline and delaying AD progression. By inhibiting platelet aggregation through irreversible blockade of cyclooxygenase-1 activity, aspirin may counteract pathological processes such as cerebral amyloid angiopathy, microinfarctions, and impaired perfusion, which are promoted by increased platelet reactivity and coagulation abnormalities and are known to contribute to dementia (Nguyen et al., 2022; Li et al., 2021; Tao et al., 2024; Ramos-Cejudo et al., 2022). Aspirin may reduce the risk of AD by targeting not only cerebrovascular dysfunction but also neuroinflammation (Cunningham and Skelly, 2012; Chandra et al., 2018). However, the relationship between aspirin use and AD dementia prevention remains inconsistent, with some studies reporting a protective effect and others showing no significant benefit (Jordan et al., 2020). The specific effect of aspirin use on the MCI-to-AD progression remains understudied, especially in large, population-based cohorts with long follow-up periods.

Analysis of different age groups is essential for assessing the role of aspirin in AD prevention. Aging is not only the strongest risk factor for AD but also profoundly affects the disease process and the pharmacokinetics of medications, including aspirin. In older adults, age-related increases in vascular comorbidities and neuroinflammation may amplify the potential benefits of aspirin, as these are key targets for its anti-inflammatory and antithrombotic actions (Livingston et al., 2020; Lopez et al., 2003). Conversely, younger individuals with MCI may experience different effects owing to a lower baseline vascular risk and different mechanisms of cognitive decline. Furthermore, the risks associated with aspirin use, such as bleeding, may outweigh its benefits in younger populations, making age-stratified analyses essential for understanding its safety and efficacy (McNeil et al., 2018).

In this nationwide cohort study, we analyzed >500,000 individuals with MCI to assess the effects of aspirin use on AD conversion risk. By focusing on these age-related differences, we aimed to provide a nuanced understanding of the role of aspirin in dementia prevention and to inform targeted therapeutic strategies for at-risk populations.

## 2 Methods

### 2.1 Study design

**Figure 1:**
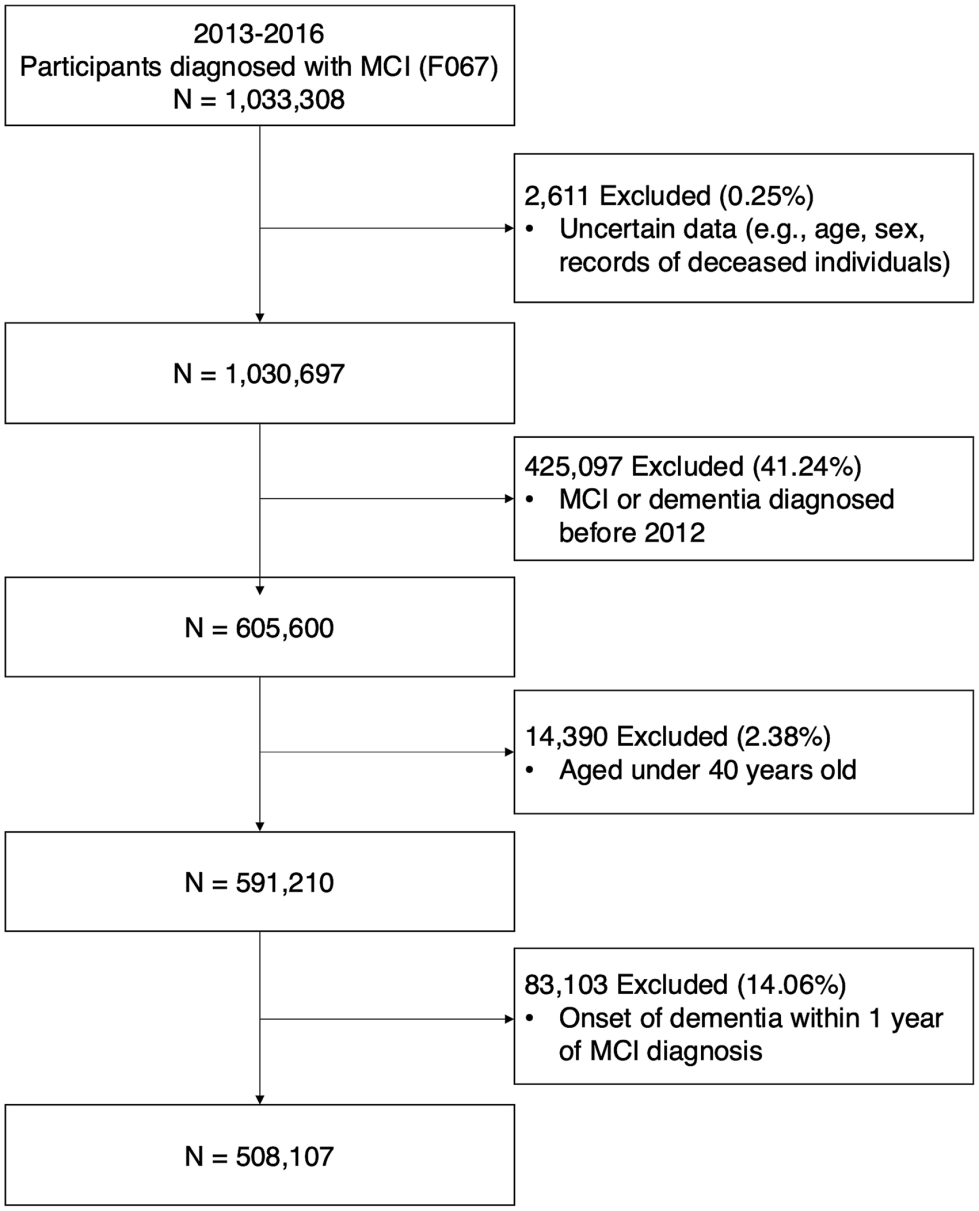
Flow diagram of study participants.

Data was sourced from a customized research database of the National Health Insurance System (NHIS). The National Health Insurance Corporation in Korea operates this mandatory health insurance system, covering >97% of South Korea’s population. The database includes demographic information, death dates, and healthcare utilization details such as diagnostic codes, prescription records, medical procedures, and treatments. Data from January 1, 2012, to December 31, 2021, were used in this study. Patients newly diagnosed with MCI between January 1, 2013, and December 31, 2016, were included. MCI was diagnosed using the International Classification of Diseases, 10th Revision (ICD-10) code F067. Individuals diagnosed with MCI before 2013, those with records of dementia (ICD-10 codes; F00-F03, G30-G31) before their MCI diagnosis, and individuals diagnosed with dementia within 1 year after their MCI diagnosis were excluded. The participants were followed up from MCI diagnosis until AD dementia occurrence, death, or the end of the study period. The primary outcome was AD dementia incidence, defined by disease codes F00 or G30, and accompanied by dementia medication prescriptions such as rivastigmine, galantamine, donepezil, or memantine (Kim et al., 2023; Kim et al., 2020). The secondary outcome was the occurrence of ischemic or hemorrhagic stroke. Ischemic and hemorrhagic stroke were defined by disease codes during at least 2 d of hospital admission (ischemic stroke, I61; hemorrhagic stroke, I63). Figure 1 shows the participant selection process.

This study was conducted in accordance with the Declaration of Helsinki and its later amendments. The study protocol was approved by the Institutional Review Board of Wonju Severance Christian Hospital (CR321308). The requirement for informed consent was formally waived owing to the retrospective study design and use of deidentified participant data.

### 2.2 Assessment of aspirin use

Aspirin use was evaluated by extracting information from the NHIS database using aspirin prescription codes (Oh and Song, 2020). The participants were divided into two groups according to their aspirin prescription history: users and nonusers. The user group included individuals prescribed aspirin at least once after MCI diagnosis. The nonuser group comprised individuals never prescribed aspirin during the study period. Additional analyses stratified by age, using 65 years as the cutoff, were conducted based on methods used in previous studies (Dunne et al., 2021; Mattke et al., 2023; Liu et al., 2023). To examine total aspirin dosage effects, the entire duration of aspirin administration in the user group was divided into quartiles. To address skewness in the duration and linearize its relationship with AD dementia risk, the variable was log-transformed (Choi et al., 2022). Using the lowest duration group (Q1) as the reference, the AD risk was evaluated in the other groups. The groups were divided into quartiles based on aspirin use duration: Q1 for 1–126 d, Q2 for 127–724 d, Q3 for 725–1,695 d, and Q4 for ≥1,696 days (Table 1).

**Table 1:** Descriptive statistics comparing patients with mild cognitive impairment with and without aspirin prescription, *n* (%).

Variables		Total MCI		Nonuser		User		
		*n*	(%)	*n*	(%)	*n*	(%)	*p*-value
Total		508,107	100.0	315,569	62.1	192,538	37.9	<0.0001
Conversion to AD dementia		39,318	7.7	16,746	8.7	22,572	7.2	<0.0001
Duration of AD conversion (y), median (IQR)				3.0 (1.6–4.4)		3.6 (2.2–5.0)		<0.0001
Aspirin duration (d), mean ± SD		–	–	–	–	977.3 ± 906.3		
Aspirin duration (Quartile, d)								<0.0001
	Q1 (1–126)	–	–	–	–	48,197	25.03	
	Q2 (127–724)	–	–	–	–	48,115	24.99	
	Q3 (725–1,695)	–	–	–	–	48,092	24.98	
	Q4 (1696–)	–	–	–	–	48,134	25.00	
Age, Mean ± SD		67.6 ± 10.7		66.4 ± 11.1		69.7 ± 9.6		<0.0001
Sex								<0.0001
	Men	168,903	33.2	96,136	30.5	72,767	37.8	
	Women	339,204	66.8	219,433	69.5	119,771	62.2	
Income								<0.0001
	Low	130,638	25.7	81,516	25.8	49,122	25.5	
	Middle	290,883	57.2	182,063	57.7	108,820	56.5	
	High	86,586	17.0	51,990	16.5	34,596	18.0	
Hypertension								<0.0001
	No	239,444	47.1	181,995	57.7	57,449	29.8	
	Yes	268,663	52.9	133,574	42.3	135,089	70.2	
Diabetes mellitus								<0.0001
	No	374,277	73.7	249,136	78.9	125,141	65.0	
	Yes	133,830	26.3	66,433	21.1	67,397	35.0	
Dyslipidemia								<0.0001
	No	243,034	47.8	170,398	54.0	72,636	37.7	
	Yes	265,073	52.2	145,171	46.0	119,902	62.3	
Heart failure								<0.0001
	No	485,772	95.6	306,319	97.1	179,453	93.2	
	Yes	22,335	4.4	9,250	2.9	13,085	6.8	
CKD								<0.0001
	No	499,804	98.4	312,110	98.9	187,694	97.5	
	Yes	8,303	1.6	3,459	1.1	4,844	2.5	
Cancer								<0.0001
	No	486,174	95.7	301,005	95.4	185,169	96.2	
	Yes	21,933	4.3	14,564	4.6	7,369	3.8	
COPD								<0.0001
	No	499,644	98.3	310,740	98.5	188,904	98.1	
	Yes	8,463	1.7	4,829	1.5	3,634	1.9	

AD, Alzheimer’s disease; SD, standard deviation; CKD, chronic kidney disease; COPD, chronic obstructive pulmonary disease; MCI, mild cognitive impairment.

### 2.3 Covariates

The study covariates included sex, income level, and chronic conditions, including hypertension, diabetes mellitus, dyslipidemia, heart failure, chronic kidney disease (CKD), cancer, and chronic obstructive pulmonary disease (COPD). Income levels were categorized into three groups based on health insurance data. Hypertension and diabetes mellitus were defined by disease codes during at least one hospital admission or at least two outpatient department visits (hypertension, I10-I13 or I15; diabetes mellitus, E11-E14). Dyslipidemia, heart failure, CKD, and cancer were defined by disease codes during at least one hospital admission or outpatient department visit (dyslipidemia, E78; heart failure, I50; CKD, N18; cancer, C00-C97 with rare intractable disease code V193). COPD was defined by codes J41-J44 during at least one hospital admission.

### 2.4 Statistical analysis

Patient baseline characteristics were evaluated using independent t-tests for continuous variables and chi-square tests for categorical variables. A Cox proportional hazards model was used to analyze the effects of aspirin. Two models with progressively increasing adjustment levels were used to account for potential confounding factors. Model 1 was adjusted for age and sex. Model 2 was further adjusted for hypertension, diabetes mellitus, dyslipidemia, heart failure, CKD, cancer, and COPD. Survival time was defined as the duration from MCI diagnosis to AD dementia onset as the primary outcome, and stroke, death, or end of the study as the secondary outcome. Furthermore, we conducted additional analyses using inverse probability of treatment weighting (IPW) to control for differential treatment likelihood. Estimates are presented as hazard ratios (HRs) and 95% confidence intervals (CIs), with *p*-values < 0.05 indicating statistical significance. All analyses were performed using the SAS software (version 9.4; SAS Institute, Cary, NC, United States).

## 3 Results

### 3.1 Patient characteristics

Between 2013 and 2016, 508,107 patients with MCI were enrolled in this study. Among them, 192,538 (37.9%) patients were prescribed aspirin. Patients prescribed aspirin had a greater prevalence of comorbidities such as hypertension, diabetes mellitus, dyslipidemia, heart failure, CKD, and COPD than that of nonusers. Cancer was the only comorbidity with a significantly higher prevalence in the nonuser group than that in the user group (Table 1).

### 3.2 MCI to AD dementia conversion risk associated with aspirin use

We performed Cox regression analysis to evaluate the risk of MCI to AD dementia conversion (Table 2). During the follow-up period, 16,746 patients (8.7%) in the user group (median follow-up: 6.44 years) and 22,572 patients (7.2%) in the nonuser group (median follow-up: 6.31 years) progressed to AD. The median time to AD dementia occurrence in the aspirin user group was 3.6 years (IQR: 2.9 years), which was longer compared with the nonuser group at 3.0 years (IQR: 2.8 years). In the overall MCI cohort, after adjusting for covariates, the AD risk was lower among aspirin users than that in nonusers (Model 2 adjusted hazard ratio [aHR] 0.939, 95% CI 0.920–0.959, *p* < 0.0001).

**Table 2:** Cox regression analysis of the association of aspirin use with dementia due to Alzheimer’s disease occurrence.

Group		Event		IR	Unadjusted			Model 1*			Model 2†		
	*N*	*n*	%		HR	95% CI	*p*-value	HR	95% CI	*p*-value	HR	95% CI	*p*-value
Total cohort													
Nonuser	315,569	22,572	7.2	11.65	1			1			1		
User	192,538	16,746	8.7	13.99	1.202	1.178–1.227	<0.0001	0.953	0.934–0.972	<0.0001	0.939	0.920–0.959	<0.0001
Age ≥ 65 years													
Nonuser	179,795	20,385	11.3	19.66	1			1			1		
User	137,883	15,489	11.2	18.73	0.952	0.933–0.972	<0.0001	0.941	0.921–0.961	<0.0001	0.934	0.914–0.955	<0.0001
Age < 65 years													
Nonuser	135,774	2,187	1.6	2.43	1			1			1		
User	54,655	1,257	2.3	3.40	1.398	1.304–1.498	<0.0001	1.144	1.067–1.228	0.0002	1.059	0.984–1.140	0.1277

*Model 1 was adjusted for age and sex. †Model 2 was further adjusted for age, sex, and comorbidities, including hypertension, diabetes mellitus, dyslipidemia, heart failure, chronic kidney disease, cancer, and chronic obstructive pulmonary disease. IR, incidence rate per 1,000 person-years; HR, hazard ratio; CI, confidence interval.

We then performed Cox regression analysis to assess the AD risk in the user group after log transformation of the total duration of aspirin use (Supplementary Table 1). Even in the covariate-adjusted models, long-term aspirin use was significantly associated with lower AD progression risk (Model 2 aHR = 0.913, 95% CI 0.907–0.920, *p* < 0.0001). Compared with the Q1 group, the AD risk increased in the Q2 group (Model 2 aHR 1.154, 95% CI 1.108–1.202, *p* < 0.0001) with longer aspirin use. However, the risk was significantly associated with a lower aHR in the Q3 group (Model 2 aHR 0.932, 95% CI 0.895–0.972, *p* = 0.009), which was further pronounced in the Q4 group (Model 2 aHR 0.357, 95% CI 0.338–0.376, *p* < 0.0001) (Supplementary Table 1).

### 3.3 Age-stratified analysis of conversion risk associated with aspirin use

Among 317,678 patients with MCI aged ≥65 years, 137,883 (43.4%) were prescribed aspirin. This group showed similar demographic trends to those observed in the overall population (Supplementary Tables 2, 3). We performed Cox regression analysis to evaluate the progression risk of MCI in each age group (Table 2). In the older group, 15,489 (11.2%) aspirin users and 20,385 (11.3%) nonusers progressed to AD during the observation period. Age-stratified analysis revealed no significant association in the younger group (Model 2 aHR 1.059, 95% CI 0.984–1.140, *p* = 0.1277), whereas a lower risk was observed in the older group (Model 2 aHR 0.934, 95% CI 0.914–0.955, *p* < 0.0001). In subgroup analyses of older patients with MCI (Supplementary Table 4), women aspirin users and individuals with dyslipidemia showed higher AD conversion risks than those in men nonusers and those without dyslipidemia, respectively (P for interaction < 0.05, Supplementary Table 4). Given the observed age-related differences in the effect of aspirin, we conducted an IPW-weighted Cox regression analysis as a sensitivity analysis (Supplementary Table 5). Following propensity score matching, the adjusted hazard ratio (Model 2) for aspirin users in the overall cohort was 0.953 (95% CI: 0.934–0.973, *p* < 0.0001), consistent with the original Cox regression results and thereby supporting the robustness of our findings.

### 3.4 Stroke occurrence risk associated with aspirin use

We also evaluated the risk of ischemic and hemorrhagic stroke associated with aspirin use using a Cox regression model (Table 3). In patients aged <65 years, aspirin use was associated with significantly reduced ischemic stroke risk (Model 2 aHR 0.891, 95% CI 0.816–0.973, *p* = 0.0099), whereas ischemic stroke risk was not reduced among older individuals. Aspirin use was associated with an increased hemorrhagic stroke risk in both groups. Notably, even after adjusting for covariates, the hemorrhagic stroke risk was significantly higher in the younger group (Model 2 aHR 5.082, 95% CI 4.838–5.338, *p* < 0.0001) compared with that in the older group (Model 2 aHR 2.726, 95% CI 2.660–2.793, *p* < 0.0001).

**Table 3:** Cox regression analysis of the association of aspirin use with stroke occurrence.

Stroke	Age group	Aspirin	Total	Event		IR	Unadjusted				Model 1*				Model 2†			
			*N*	*n*	(%)		HR	95% CI		*p*-value	HR	95% CI		*p*-value	HR	95% CI		*p*-value
Ischemic stroke	Total	No	315,569	3,927	1.24	1.97	1				1				1			
		Yes	192,538	2,957	1.54	2.40	1.219	1.163	1.279	<0.0001	1.13	1.077	1.186	<0.0001	0.975	0.927	1.025	0.3190
	≥65	No	179,795	2,305	1.28	2.11	1				1				1			
		Yes	137,883	2098	1.52	2.43	1.154	1.088	1.224	<0.0001	1.12	1.055	1.188	0.0002	1.044	0.982	1.110	0.1658
	<65	No	135,774	1,622	1.19	1.80	1				1				1			
		Yes	54,655	859	1.57	2.32	1.300	1.197	1.412	<0.0001	1.205	1.107	1.311	<0.0001	0.891	0.816	0.973	0.0099
Hemorrhagic stroke	Total	No	315,569	12,590	3.99	6.40	1				1				1			
		Yes	192,538	28,879	15.00	25.23	3.917	3.835	3.999	<0.0001	3.356	3.286	3.427	<0.0001	3.094	3.027	3.163	<0.0001
	≥65	No	179,795	10,117	5.63	9.45	1				1				1			
		Yes	137,883	22,533	16.34	28.23	2.979	2.91	3.05	<0.0001	2.899	2.831	2.968	<0.0001	2.726	2.66	2.793	<0.0001
	<65	No	135,774	2,473	1.82	2.76	1				1				1			
		Yes	54,655	6,346	11.61	18.33	6.588	6.289	6.902	<0.0001	5.877	5.605	6.162	<0.0001	5.082	4.838	5.338	<0.0001

*Model 1 was adjusted for age and sex. †Model 2 was further adjusted for age, sex, and comorbidities, including hypertension, diabetes mellitus, dyslipidemia, heart failure, chronic kidney disease, cancer, and chronic obstructive pulmonary disease. Abbreviations: IR, incidence rate per 1,000 person-years; HR, hazard ratio; CI, confidence interval.

## 4 Discussion

In this nationwide study, we identified that aspirin use was associated with a reduced risk of AD conversion in patients with MCI. This protective effect increased with the longer duration of aspirin use and was most pronounced in men aged ≥65 years.

Cognitive decline occurs and progresses over time (Livingston et al., 2020). The rate of progression through the continuum from subjective cognitive decline to MCI, and eventually to dementia, is widely known to vary depending on individual risk factors (Mitchell and Shiri-Feshki, 2009; Dunne et al., 2021; Liu et al., 2023). Delaying progression to dementia at this stage can substantially reduce individual disease burden and societal costs (Livingston et al., 2020; Gauthier et al., 2006). Previous studies have consistently identified cardiovascular disease (CVD) as a risk factor for AD (Lopez et al., 2003). Aspirin, which is commonly used for the primary and secondary prevention of CVD, has been extensively investigated for its association with the risk of cognitive decline (Zheng and Roddick, 2019; Ding et al., 2023). Despite numerous studies, the effect of aspirin use on AD occurrence remains controversial, and no large-scale cohort study has investigated the role of aspirin in MCI-to-AD progression (Jordan et al., 2020).

In this study, we analyzed a nationwide cohort of >500,000 patients with MCI, with 37.9% of them prescribed aspirin during a follow-up of up to 9 years. This prescription rate exceeded previously reported rates, likely reflecting the relatively high prevalence of cardiovascular and cerebrovascular diseases in patients with MCI (Oh and Song, 2020; Yoo et al., 2024). In this study, aspirin users had a higher overall comorbidity burden than that in nonusers, with notable differences in conditions that increased CVD risk, such as hypertension, diabetes mellitus, and dyslipidemia (Lopez et al., 2003).

Survival analysis revealed that aspirin use was associated with reduced AD dementia risk. Because comorbidities can act as risk factors, we conducted adjusted analyses, observing consistent trends. To account for potential variation in the effects based on the duration of aspirin use, we analyzed the risk of AD dementia occurrence according to the duration of aspirin administration. Log transformation and Cox regression analysis confirmed that prolonged aspirin use was associated with reduced AD dementia risk. Similarly, in the quartile-based analysis of aspirin use duration, the Q4 group showed the greatest reduction in the progression risk of MCI. While previous randomized controlled trials concluded that no association exists between aspirin use and AD risk (Jordan et al., 2020; McNeil et al., 2018; ASPREE Investigator Group, 2013), our findings suggest that this lack of association might result from insufficient follow-up periods.

Based on previous studies suggesting that MCI-to-AD progression varies by age, we divided the cohort into two groups using 65 years as the cutoff age (Livingston et al., 2020; Dunne et al., 2021; Mattke et al., 2023). The protective effect of aspirin was particularly pronounced in older individuals (Nilsson et al., 2003). This effect may be attributed to age-related differences in aspirin pharmacokinetics or more pronounced anti-inflammatory effects in older individuals (McNeil et al., 2018; ASPREE Investigator Group, 2013; Grosser et al., 2006). Furthermore, age-related alterations in platelet reactivity and hemostatic balance may also contribute to the differential impact of aspirin in older adults, supporting the biological plausibility of its protective role in MCI-to-AD progression (Nguyen et al., 2022; Ramos-Cejudo et al., 2022; Poscablo et al., 2024). To account for the effects of covariates, a subgroup analysis was conducted in the older age group. Excluding sex, only dyslipidemia yielded statistically significant results, exhibiting a more pronounced HR reduction in unaffected individuals. The apolipoprotein E4 allele, a known AD risk factor, is involved in dyslipidemia (McGrattan et al., 2022; Yesiltepe et al., 2024; Husain et al., 2021). Patients with dyslipidemia may be more susceptible to the action of the apolipoprotein, which could reduce the effect of aspirin on MCI-to-AD conversion, potentially explaining these findings (Ding et al., 2023).

Aspirin is one of the most commonly used medications for the secondary prevention of ischemic stroke. However, owing to its cyclooxygenase enzyme inhibitory effect, it can increase the risk of hemorrhagic stroke (McNeil et al., 2018; Zheng and Roddick, 2019). In this study, aspirin use was associated with an elevated hemorrhagic stroke risk across all age groups, particularly in younger patients. Regarding ischemic stroke, a significant reduction in risk was observed only in younger individuals. Combined with the age-based analysis of AD dementia risk, these findings suggested that aspirin use may help delay the MCI-to-AD progression in older adults when the risk of hemorrhagic stroke is carefully managed. However, in younger patients, the significantly increased HR for hemorrhagic stroke and lack of a meaningful reduction in AD risk indicated that aspirin use offers limited benefits in this population. Similar to previous studies suggesting that aspirin use in patients with Alzheimer’s disease may increase the risk of intracerebral hemorrhage without necessarily leading to cognitive deterioration, clinicians should carefully assess the indication for aspirin and not hesitate to use it when appropriate (Thoonsen et al., 2010). Careful patient selection and risk–benefit evaluation remain essential to ensure the safe and effective use of aspirin in this population.

The present study had several limitations. First, AD dementia diagnosis relied on diagnostic codes and antidementia medication prescriptions. Although this methodology reduces the likelihood of misclassification, it does not eliminate the possibility of errors. Furthermore, comorbid conditions were also identified using diagnostic codes from claims data, which might have introduced misclassification bias. Since the primary focus of this study was to evaluate the effect of aspirin use on the risk of progression from MCI to AD dementia, we did not include other causes of dementia in our analysis. To comprehensively assess the potential impact of aspirin, future studies should consider including various types of dementia beyond AD. Second, the study lacked comprehensive clinical data on dementia. Specifically, information on cognitive assessments, neuropsychological testing, detailed clinical symptomatology, AD biomarker profiles, and noncognitive manifestations, including behavioral and psychological symptoms, was not included. Third, the study did not account for the use of other antiplatelet medications, such as clopidogrel, which could have confounded the observed associations. Due to limitations in the available data, adjustment for these agents was not possible and should be considered in future research. Fourth, because aspirin is available over the counter in Korea, some use may not have been captured in the prescription claims data, potentially leading to exposure misclassification. Furthermore, the absence of a trial emulation design precludes the assessment of treatment adherence over time, which may have influenced the observed associations.

This nationwide study revealed that aspirin use is associated with a reduced risk of AD conversion in patients with MCI. This protective effect increased with prolonged aspirin use and was most evident in older men. However, as aspirin use may increase the risk of bleeding, including hemorrhagic stroke, personalized treatment tailored to individual patients is necessary. Further studies are required to elucidate the precise mechanisms underlying this process.

## Data Availability

The datasets presented in this article are not readily available because the original NHIS data is not owned by the researchers, and Korean law prohibits the transfer of data files to any third party. Requests to access the datasets should be directed to the data were used after obtaining approval from the Institutional Review Board and the Korea NHIS Big Data Operations Department (https://nhiss.nhis.or.kr/bd/ay/bdaya001iv.do).
