# Supplementary material for: Effect of aspirin use on conversion risk from mild cognitive impairment to Alzheimer’s disease
Source: Front Aging Neurosci. 2025 Aug 6;17:1603892. doi: 10.3389/fnagi.2025.1603892 (PMC12364806; doi:10.3389/fnagi.2025.1603892)
Supplement: Supplementary file 1 [file Table_1.docx]

**Supplementary Table 1. Cox regression analysis of Alzheimer’s disease dementia risk by aspirin administration duration in patients with mild cognitive impairment**

|  | **Total aspirin administration duration** |  | **Event** | |  | **Unadjusted** | | | | **Model 1*** | | | | **Model 2†** | | |  |
| --- | --- | --- | --- | --- | --- | --- | --- | --- | --- | --- | --- | --- | --- | --- | --- | --- | --- |
|  |  | **N** | **n** | **(%)** | **IR** | **HR** | **95% CI** | **p-value** | **HR** | | **95% CI** | **p-value** | **HR** | | **95% CI** | **p-value** | |
| Log (Total aspirin duration) | | 192 538 | 16 746 | 8.7 | 13.99 | 0.935 | 0.929–0.942 | <.0001 | 0.921 | | 0.914–0.927 | <.0001 | 0.913 | | 0.907–0.920 | <.0001 | |
| Quartile groups (days) | |  |  |  |  |  |  |  |  | |  |  |  | |  |  | |
|  | Q1 (1–126) | 48 197 | 4339 | 9.0 | 15.11 | 1 |  |  | 1 | |  |  | 1 | |  |  | |
|  | Q2 (127–724) | 48 115 | 5369 | 11.2 | 19.41 | 1.297 | 1.246–1.350 | <.0001 | 1.188 | | 1.142–1.237 | <.0001 | 1.154 | | 1.108–1.202 | <.0001 | |
|  | Q3 (725–1695) | 48 092 | 5017 | 10.4 | 16.82 | 1.107 | 1.063–1.153 | <.0001 | 0.974 | | 0.935–1.014 | 0.1996 | 0.932 | | 0.895–0.972 | 0.0009 | |
|  | Q4 (≥ 1696) | 48 134 | 2021 | 4.2 | 6.04 | 0.393 | 0.373–0.414 | <.0001 | 0.374 | | 0.355–0.394 | <.0001 | 0.357 | | 0.338–0.376 | <.0001 | |

* Model 1 adjusted for age and sex.

† Model 2 was further adjusted for age, sex, and comorbidities, including hypertension, diabetes mellitus, dyslipidemia, heart failure, chronic kidney disease, cancer, and chronic obstructive pulmonary disease.

Abbreviations: IR, incidence rate per 1,000 person-years; HR, hazard ratio; CI, confidence interval

**Supplementary Table 2. Descriptive statistics comparing patients with MCI with and without an aspirin prescription over 65 years old, *n* (%)**

|  |  | **Total MCI** | | **Nonuser** | | **User** | |  |
| --- | --- | --- | --- | --- | --- | --- | --- | --- |
|  |  | **n** | **(%)** | **n** | **(%)** | **n** | **(%)** | ***p*-value** |
| Total |  | 317 678 | 100.0 | 179 795 | 56.6 | 137 883 | 43.4 |  |
| Age, *Mean* (SD) |  | 74.41 (6.31) |  | 74.35 (6.48) |  | 74.49 (6.08) |  | <.0001 |
| Sex |  |  |  |  |  |  |  | <.0001 |
|  | Male | 106 422 | 33.5 | 56 178 | 31.2 | 50 244 | 36.4 |  |
|  | Female | 211 256 | 66.5 | 123 617 | 68.8 | 87 639 | 63.6 |  |
| Income |  |  |  |  |  |  |  | 0.0003 |
|  | Low | 78 501 | 24.7 | 44 721 | 24.9 | 33 780 | 24.5 |  |
|  | Middle | 174 327 | 54.9 | 98 796 | 54.9 | 75 531 | 54.8 |  |
|  | High | 64 850 | 20.4 | 36 278 | 20.2 | 28 572 | 20.7 |  |
| Hypertension |  |  |  |  |  |  |  | <.0001 |
|  | No | 119 413 | 37.6 | 85 030 | 47.3 | 34 383 | 24.9 |  |
|  | Yes | 198 265 | 62.4 | 94 765 | 52.7 | 103 500 | 75.1 |  |
| Diabetes mellitus |  |  |  |  |  |  |  | <.0001 |
|  | No | 220 895 | 69.5 | 133 837 | 74.4 | 87 058 | 63.1 |  |
|  | Yes | 96 783 | 30.5 | 45 958 | 25.6 | 50 825 | 36.9 |  |
| Dyslipidemia |  |  |  |  |  |  |  | <.0001 |
|  | No | 144 058 | 45.3 | 92 513 | 51.5 | 51 545 | 37.4 |  |
|  | Yes | 173 620 | 54.7 | 87 282 | 48.5 | 86 338 | 62.6 |  |
| Heart failure |  |  |  |  |  |  |  | <.0001 |
|  | No | 299 148 | 94.2 | 172 224 | 95.8 | 126 924 | 92.1 |  |
|  | Yes | 18 530 | 5.8 | 7571 | 4.2 | 10 959 | 7.9 |  |
| CKD |  |  |  |  |  |  |  | <.0001 |
|  | No | 311 243 | 98.0 | 177 103 | 98.5 | 134 140 | 97.3 |  |
|  | Yes | 6435 | 2.0 | 2692 | 1.5 | 3743 | 2.7 |  |
| Cancer |  |  |  |  |  |  |  | <.0001 |
|  | No | 302 274 | 95.2 | 170 157 | 94.6 | 132 117 | 95.8 |  |
|  | Yes | 15 404 | 4.8 | 9638 | 5.4 | 5766 | 4.2 |  |
| COPD |  |  |  |  |  |  |  | 0.0617 |
|  | No | 311 085 | 97.9 | 176 138 | 98.0 | 134 947 | 97.9 |  |
|  | Yes | 6593 | 2.1 | 3657 | 2.0 | 2936 | 2.1 |  |

Abbreviations: MCI, mild cognitive impairment; SD, standard deviation; CKD, chronic kidney disease; COPD, chronic obstructive pulmonary disease

**Supplementary Table 3. Descriptive statistics comparing patients with MCI with and without an aspirin prescription under 65 years old, *n* (%)**

|  |  | **Total MCI** | | **Nonuser** | | **User** | |  |
| --- | --- | --- | --- | --- | --- | --- | --- | --- |
|  |  | **n** | **(%)** | **n** | **(%)** | **n** | **(%)** | ***p*-value** |
| Total |  | 190 429 | 100.0 | 135 774 | 71.3 | 54 655 | 28.7 |  |
| Age, *Mean* (SD) |  | 56.28 (5.84) |  | 55.75 (5.99) |  | 57.57 (5.22) |  | <.0001 |
| Sex |  |  |  |  |  |  |  | <.0001 |
|  | Male | 62 481 | 32.8 | 39 958 | 29.4 | 22 523 | 41.2 |  |
|  | Female | 127 948 | 67.2 | 95 816 | 70.6 | 32 132 | 58.8 |  |
| Income |  |  |  |  |  |  |  | <.0001 |
|  | Low | 52 137 | 27.4 | 36 795 | 27.1 | 15 342 | 28.1 |  |
|  | Middle | 116 556 | 61.2 | 83 267 | 61.3 | 33 289 | 60.9 |  |
|  | High | 21 736 | 11.4 | 15 712 | 11.6 | 6024 | 11.0 |  |
| Hypertension |  |  |  |  |  |  |  | <.0001 |
|  | No | 120 031 | 63.0 | 96 965 | 71.4 | 23 066 | 42.2 |  |
|  | Yes | 70 398 | 37.0 | 38 809 | 28.6 | 31 589 | 57.8 |  |
| Diabetes mellitus |  |  |  |  |  |  |  | <.0001 |
|  | No | 153 382 | 80.5 | 115 299 | 84.9 | 38 083 | 69.7 |  |
|  | Yes | 37 047 | 19.5 | 20 475 | 15.1 | 16 572 | 30.3 |  |
| Dyslipidemia |  |  |  |  |  |  |  | <.0001 |
|  | No | 98 976 | 52.0 | 77 885 | 57.4 | 21 091 | 38.6 |  |
|  | Yes | 91 453 | 48.0 | 57 889 | 42.6 | 33 564 | 61.4 |  |
| Heart failure |  |  |  |  |  |  |  | <.0001 |
|  | No | 186 624 | 98.0 | 134 095 | 98.8 | 52 529 | 96.1 |  |
|  | Yes | 3805 | 2.0 | 1679 | 1.2 | 2126 | 3.9 |  |
| Chronic kidney disease |  |  |  |  |  |  |  | <.0001 |
|  | No | 188 561 | 99.0 | 135 007 | 99.4 | 53 554 | 98.0 |  |
|  | Yes | 1868 | 1.0 | 767 | 0.6 | 1101 | 2.0 |  |
| Cancer |  |  |  |  |  |  |  | <.0001 |
|  | No | 183 900 | 96.6 | 130 848 | 96.4 | 53 052 | 97.1 |  |
|  | Yes | 6529 | 3.4 | 4926 | 3.6 | 1603 | 2.9 |  |
| COPD |  |  |  |  |  |  |  | <.0001 |
|  | No | 188 559 | 99.0 | 134 602 | 99.1 | 53 957 | 98.7 |  |
|  | Yes | 1870 | 1.0 | 1172 | 0.9 | 698 | 1.3 |  |

Abbreviations: MCI, mild cognitive impairment; SD, standard deviation; COPD, chronic obstructive pulmonary disease

**Supplementary Table 4. Hazard ratios with 95% confidence intervals of the AD conversion risk in older individuals with aspirin use in the subgroup analysis**

| **Subgroup** |  | **Aspirin** | **N** | **Event (AD)** | **Duration** | **IR** | **HR** | **95% CI** | | **P for interaction** |
| --- | --- | --- | --- | --- | --- | --- | --- | --- | --- | --- |
| Sex |  |  |  |  |  |  |  |  |  | 0.0049 |
|  | Male | Nonuser | 56 178 | 5638 | 303 952 | 18.55 | 1 |  |  |  |
|  |  | User | 50 244 | 4909 | 288 434 | 17.02 | 0.905 | 0.870 | 0.942 |  |
|  | Female | Nonuser | 123 617 | 14 747 | 733 128 | 20.12 | 1 |  |  |  |
|  |  | User | 87 639 | 10 580 | 538 362 | 19.65 | 0.943 | 0.919 | 0.967 |  |
| Income |  |  |  |  |  |  |  |  |  | 0.3717 |
|  | Low | Nonuser | 44 721 | 5475 | 253 778 | 21.57 | 1 |  |  |  |
|  |  | User | 33 780 | 4188 | 199 246 | 21.02 | 0.953 | 0.914 | 0.993 |  |
|  | Middle | Nonuser | 98 796 | 10 785 | 572 867 | 18.83 | 1 |  |  |  |
|  |  | User | 75 531 | 8066 | 455 479 | 17.71 | 0.924 | 0.896 | 0.952 |  |
|  | High | Nonuser | 36 278 | 4125 | 210 436 | 19.60 | 1 |  |  |  |
|  |  | User | 28 572 | 3235 | 172 071 | 18.80 | 0.932 | 0.889 | 0.978 |  |
| Hypertension |  |  |  |  |  |  |  |  |  | 0.1394 |
|  | No | Nonuser | 85 030 | 9394 | 502 348 | 18.70 | 1 |  |  |  |
|  |  | User | 34 383 | 3597 | 211 802 | 16.98 | 0.909 | 0.875 | 0.945 |  |
|  | Yes | Nonuser | 94 765 | 10 991 | 534 733 | 20.55 | 1 |  |  |  |
|  |  | User | 103 500 | 11 892 | 614 993 | 19.34 | 0.942 | 0.918 | 0.967 |  |
| Diabetes mellitus |  |  |  |  |  |  |  |  |  | 0.1892 |
|  | No | Nonuser | 133 837 | 14 841 | 783 977 | 18.93 | 1 |  |  |  |
|  |  | User | 87 058 | 9510 | 530 337 | 17.93 | 0.942 | 0.918 | 0.968 |  |
|  | Yes | Nonuser | 45 958 | 5544 | 253 103 | 21.90 | 1 |  |  |  |
|  |  | User | 50 825 | 5979 | 296 459 | 20.17 | 0.912 | 0.879 | 0.947 |  |
| Dyslipidemia |  |  |  |  |  |  |  |  |  | 0.0138 |
|  | No | Nonuser | 92 513 | 11 272 | 530 652 | 21.24 | 1 |  |  |  |
|  |  | User | 51 545 | 6207 | 308 994 | 20.09 | 0.910 | 0.882 | 0.940 |  |
|  | Yes | Nonuser | 87 282 | 9113 | 506 428 | 17.99 | 1 |  |  |  |
|  |  | User | 86 338 | 9282 | 517 802 | 17.93 | 0.951 | 0.923 | 0.980 |  |
| Heart failure |  |  |  |  |  |  |  |  |  | 0.0583 |
|  | No | Nonuser | 172 224 | 19 366 | 1 000 526 | 19.36 | 1 |  |  |  |
|  |  | User | 126 924 | 14 063 | 768 537 | 18.30 | 0.934 | 0.913 | 0.955 |  |
|  | Yes | Nonuser | 7571 | 1019 | 36 555 | 27.88 | 1 |  |  |  |
|  |  | User | 10 959 | 1426 | 58 259 | 24.48 | 0.898 | 0.827 | 0.974 |  |
| Chronic kidney disease | |  |  |  |  |  |  |  |  | 0.8885 |
|  | No | Nonuser | 177 103 | 20 082 | 1 025 587 | 19.58 | 1 |  |  |  |
|  |  | User | 134 140 | 15 057 | 809 448 | 18.60 | 0.932 | 0.912 | 0.953 |  |
|  | Yes | Nonuser | 2692 | 303 | 11 493 | 26.36 | 1 |  |  |  |
|  |  | User | 3743 | 432 | 17 347 | 24.90 | 0.920 | 0.793 | 1.069 |  |
| Cancer |  |  |  |  |  |  |  |  |  | 0.4167 |
|  | No | Nonuser | 170 157 | 19 561 | 991 936 | 19.72 | 1 |  |  |  |
|  |  | User | 132 117 | 14 953 | 794 887 | 18.81 | 0.934 | 0.914 | 0.955 |  |
|  | Yes | Nonuser | 9638 | 824 | 45 144 | 18.25 | 1 |  |  |  |
|  |  | User | 5766 | 536 | 31 908 | 16.80 | 0.881 | 0.787 | 0.986 |  |
| Chronic obstructive pulmonary disease | | |  |  |  |  |  |  |  | 0.5702 |
|  | No | Nonuser | 176 138 | 19 948 | 1 022 560 | 19.51 | 1 |  |  |  |
|  |  | User | 134 947 | 15 100 | 812 865 | 18.58 | 0.932 | 0.912 | 0.953 |  |
|  | Yes | Nonuser | 3657 | 437 | 14 521 | 30.09 | 1 |  |  |  |
|  |  | User | 2936 | 389 | 13 931 | 27.92 | 0.930 | 0.807 | 1.072 |  |

Abbreviations: IR, incidence rate per 1,000 person-years; HR, hazard ratio; CI, confidence interval; AD, Alzheimer’s disease

**Supplementary Table 5. Cox regression analysis of the association of aspirin uses Alzheimer’s occurrence after Propensity score matching**

|  |  | | **Unadjusted** | | | | **Model 1*** | | | | | | | **Model 2†** | | | | | |  |
| --- | --- | --- | --- | --- | --- | --- | --- | --- | --- | --- | --- | --- | --- | --- | --- | --- | --- | --- | --- | --- |
|  |  | | **HR** | **95% CI** | | ***p-value*** | | **HR** | **95% CI** | | | ***p-value*** | | | **HR** | **95% CI** | | | ***p-value*** | |
| Total cohort | | |  |  |  | |  | | |  |  | |  | | | |  |  | |  |
|  | | Nonuser | 1 |  | |  | | 1 |  | | |  | | | 1 |  | | |  | |
|  | | User | 1.202 | 1.178-1.227 | | <.0001 | | 0.969 | 0.95-0.989 | | | 0.0022 | | | 0.953 | 0.934-0.973 | | | <.0001 | |
| Age ≥ 65 years | | |  |  | |  | |  |  | | |  | | |  |  | | |  | |
|  | | Nonuser | 1 |  | |  | | 1 |  | | |  | | | 1 |  | | |  | |
|  | | User | 0.952 | 0.933-0.972 | | <.0001 | | 0.941 | 0.921-0.961 | | | <.0001 | | | 0.934 | 0.914-0.955 | | | <.0001 | |
| Age < 65 years | | |  |  | |  | |  |  | | |  | | |  |  | | |  | |
|  | | Nonuser | 1 |  | |  | | 1 |  | | |  | | | 1 |  | | |  | |
|  | | User | 1.398 | 1.304-1.498 | | <.0001 | | 1.144 | 1.067-1.228 | | | 0.0002 | | | 1.059 | 0.984-1.140 | | | 0.1279 | |

*** Model 1 was adjusted for age and sex.**

**† Model 2 was further adjusted for age, sex, and comorbidities, including hypertension, diabetes mellitus, dyslipidemia, heart failure, chronic kidney disease, cancer, and chronic obstructive pulmonary disease.**
